# Novel Regulator of Acylated Ghrelin, CF801, Reduces Weight Gain, Rebound Feeding after a Fast, and Adiposity in Mice

**DOI:** 10.3389/fendo.2015.00144

**Published:** 2015-09-25

**Authors:** Martin K. Wellman, Zachary R. Patterson, Harry MacKay, Joseph E. Darling, Bharath K. Mani, Jeffrey M. Zigman, James L. Hougland, Alfonso Abizaid

**Affiliations:** ^1^Department of Neuroscience, Carleton University, Ottawa, ON, Canada; ^2^Department of Chemistry, Syracuse University, Syracuse, NY, USA; ^3^Department of Internal Medicine, Division of Hypothalamic Research, The University of Texas Southwestern Medical Center, Dallas, TX, USA; ^4^Department of Internal Medicine, Division of Endocrinology and Metabolism, The University of Texas Southwestern Medical Center, Dallas, TX, USA; ^5^Department of Psychiatry, The University of Texas Southwestern Medical Center, Dallas, TX, USA

**Keywords:** ghrelin, ghrelin-*O*-acyltransferase, ghrelin antagonist, weight loss, appetite, energy expenditure, adiposity

## Abstract

Ghrelin is a 28 amino acid hormonal peptide that is intimately related to the regulation of food intake and body weight. Once secreted, ghrelin binds to the growth hormone secretagogue receptor-1a, the only known receptor for ghrelin and is capable of activating a number of signaling cascades, ultimately resulting in an increase in food intake and adiposity. Because ghrelin has been linked to overeating and the development of obesity, a number of pharmacological interventions have been generated in order to interfere with either the activation of ghrelin or interrupting ghrelin signaling as a means to reducing appetite and decrease weight gain. Here, we present a novel peptide, CF801, capable of reducing circulating acylated ghrelin levels and subsequent body weight gain and adiposity. To this end, we show that IP administration of CF801 is sufficient to reduce circulating plasma acylated ghrelin levels. Acutely, intraperitoneal injections of CF801 resulted in decreased rebound feeding after an overnight fast. When delivered chronically, they decreased weight gain and adiposity without affecting caloric intake. CF801, however, did cause a change in diet preference, decreasing preference for a high-fat diet and increasing preference for regular chow diet. Given the complexity of ghrelin receptor function, we propose that CF801, along with other compounds that regulate ghrelin secretion, may prove to be a beneficial tool in the study of the ghrelin system, and potential targets for ghrelin-based obesity treatments without altering the function of ghrelin receptors.

## Introduction

One potential target for the control of body weight and appetite is the gut–brain peptide ghrelin. Ghrelin, a 28-amino-acid peptide produced in the X/A-like cells of the gastric oxyntic mucosa lining the stomach, is a key regulator of both short- and long-term energy homeostasis (1–3). Circulating ghrelin levels rise in anticipation of a meal and subside once the organism is satiated (4–7). Furthermore, ghrelin has been shown to regulate several physiological processes, including glucose metabolism (8–12), insulin secretion (8, 13, 14), gastric emptying (15), cell proliferation (16, 17), memory (18), stress (19–22), anxiety (18, 19, 22–25), and reward (26–29). Acutely, ghrelin promotes food intake through interactions with a subset of distinct first order hypothalamic nuclei (2, 3, 30–34) and injections of ghrelin result in vigorous feeding bouts (35–37). Ghrelin contributes to long-term energy homeostasis by increasing body weight and adiposity, presumably through a reduction of lipid oxidation (3, 21, 38, 39). Ghrelin increases motivation to obtain food (27–29, 40) and also increases the preference for highly palatable foods by acting in the VTA (28, 29, 41, 42).

The ability of ghrelin to carry out its physiological processes relies on its affinity for the growth hormone secretagogue receptor-1a (GHSR-1a), the only known ghrelin receptor. GHSR-1a is widely expressed in both rodents and humans, with highest expression found in the hypothalamus and pituitary gland, both areas heavily invested in energy homeostasis and growth hormone secretion (43). Within the hypothalamus, GHSR-1a expression is most concentrated to the arcuate nucleus (43–45), a region important in the regulation of food intake, metabolism and energy homeostasis (3, 46, 47). Interestingly, activation of GHSR-1a requires a post-translational modification of the proghrelin peptide (1), a characteristic unique to ghrelin amongst all other metabolically active hormones. Specifically, ghrelin must be acylated on the hydroxyl group of the third serine (Ser3) residue with an *n*-octanoic acid, or other medium-chain fatty acid (MCFA) containing 6–12 carbons, in order to bind to and activate GHSR-1a (1). Modification with an *n*-octanoyl group induces a conformational flexibility that accommodates the geometric specificity of GHSR-1a’s binding pocket (48).

The enzyme responsible for the acylation of the mature ghrelin peptide is called ghrelin-*O*-acyltransferase, or GOAT (49, 50), which belongs to a superfamily of enzymes known as the membrane-bound*-O-*acyltransferases (MBOATs) (51). GOAT expression has been demonstrated in many rat, mouse, and human tissues, including the ghrelin producing X/A-like cells of the gastric oxyntic mucosa lining the stomach (52–54). To date, GOAT is the only known enzyme capable of acylating, and therefore activating ghrelin, as evidenced by structure–activity analyses of ghrelin mimetic substrates and inhibitors as well as by studies demonstrating the complete absence of acylated ghrelin in GOAT null mice (49, 55). As such, GOAT has become a target for the development of drugs to curb appetite and reduce weight gain via a reduction in acylated ghrelin concentrations (55–59). Indeed, a number of compounds that reduce GOAT activity are successful in decreasing weight gain and adiposity. For instance, daily treatment with GO-CoA-TAT, a peptide GOAT inhibitor, was effective in reducing weight gain and adiposity in mice while decreasing acylated ghrelin concentrations (60).

In this paper, we present CF801, a synthetic peptide that was thought to be a novel GOAT inhibitor, and one that decreases plasma concentrations of acylated ghrelin *in vitro* and *in vivo*. While we demonstrate that CF801 is not an efficient inhibitor of GOAT octanoylation activity, CF801 produced a number of important metabolic effects, including a decrease in weight gain and adiposity associated with changes in appetite and energy expenditure associated with decreased ghrelin concentrations.

## Materials and Methods

### Design of CF801

An approach was undertaken to design a novel GOAT inhibitor, resulting in a compound we named CF801. The first five amino acids of CF801 derive from studies carried out in 2008 by Yang et al., who examined the inhibitory effect of certain pentapeptides (e.g., GSAFL-NH_2_) on GOAT using a microsomal assay (56). While these peptides showed inhibitory activity on GOAT, it was unlikely that the peptide could effectively cross membranes to reach the endoplasmic reticulum lumen, where GOAT performs its enzymatic reactions (50). In synthesizing CF801, we extended and modified the original GSAFL-NH_2_ sequence with the addition of five more amino acids that correspond to amino acids 6–10 of the full-length ghrelin peptide. The addition of these amino acids increases the similarity of CF801 to the non-acylated ghrelin peptide sequence as well as provides an appropriate spacer for attachment of additional modifications. The second modification from the GSAFL-NH_2_ peptide was the addition of an HIV trans-activator of transcription (Tat) sequence. CF801 contains the following Tat sequence: RKKRRQRRR, with an amide group retained on the C-terminus.

The full sequence of CF801 is as follows: GSAFLSPEHQRK KRRQRRR-NH_2_, where the N-terminus has the standard free amine group (–NH_2_). Variants of this molecule can be synthesized through the inclusion of (or perhaps exclusion of) additional amino acids from the mature ghrelin protein molecule and/or using variants of the Tat sequence or other cell-penetrating peptides. CF801 was custom synthesized by Peptides International (Louisville, KY, USA).

### Cell-based ghrelin secretion assay

Ghrelin secretion studies were performed in stomach-derived ghrelinoma (SG-1) cells, cultured as described previously (61, 62). Cells were plated at a density of 5 × 10^4^ cells/mL/well on to 24-well plates pre-coated with poly-d-lysine (day 0). On day 2, the cells were treated with the indicated concentrations of test compound CF801, 24 h ahead of the test period and with 50 μM sodium octanoate–bovine serum albumin (BSA), 16 h ahead of the test period. On day 3, the medium was aspirated and the cells were treated with the same concentrations of CF801 in 500 μL serum-free DMEM (Life Technologies, Grand Island, NY, USA) supplemented with 5 mM glucose and 50 μM sodium octanoate–BSA. After a 6-h incubation, the medium was collected, placed on ice, and immediately centrifuged at 800 × *g* for 5 min. Hydrochloric acid was added to the supernatant to achieve a final concentration of 0.1 N (for stabilization of acyl ghrelin) and stored at −80°C until analysis. Assay for acyl ghrelin and total were performed using commercial ELISA kits (EMD Millipore Corporation, Billerica, MA, USA) according to the manufacturer’s instructions and read in a PowerWave XS Microplate spectrophotometer (BioTek Instruments, Inc., Winooski, VT, USA).

### Fluorescence-based GOAT activity assay

The effects of CF801 on GOAT activity were examined using a previously validated fluorescence-based assay, and in comparison with octanoyl-(Dap3)-ghrelin (1–5)–NH_2_, a well characterized GOAT inhibitor (56, 63). In this assay, membrane fractions from Sf9 insect cells were transfected to express either human (hGOAT) or mouse GOAT (mGOAT). Membranes fractions were thawed on ice and passed through an 18-gage needle 10 times enriched using a previously described method (63). Assays were performed with 20–30 μg membrane protein (for hGOAT) and 60–80 μg membrane protein (for mGOAT), 1.5 μM GSSFLC_AcDan_ peptide substrate, 500 μM octanoyl-CoA, 0–10 μM CF801, octanoyl-(Dap3)-ghrelin (1–5)–NH_2_, or carrier control (50 mM HEPES pH = 7.0 or DMSO) and 50 mM HEPES pH 7.0 in a total volume of 50 μL. All components with the exception of the acrylodan-labeled peptide substrate were incubated in the reaction vessel for 30 min prior to assay initiation. Assays were initiated by addition of the acrylodanylated peptide substrate GSSFLC_AcDan_. Assays were then incubated at room temperature for 1 h and stopped by addition of 50 μL of 20% acetic acid in isopropanol. Assays were analyzed by reverse phase HPLC with fluorescence detection. Chromatogram analysis and integration of peptide substrate and product peaks were performed using Chemstation for LC (Agilent Technologies). The trials were run in triplicate, with the % activity calculated as the integrated fluorescence intensity of the octanoylated product in the CF801 or octanoyl-(Dap3)-ghrelin (1–5)–NH_2_ run normalized by the same quantity for the vehicle-only reaction.

### *In Vivo* animal studies

#### Animals

In general, male C57BL/6J mice (The Jackson Laboratory, Bar Harbor, ME, USA) weighing 20–25 g were used as experimental subjects. Throughout the duration of the studies, mice were housed under standard laboratory conditions and received *ad libitum* access to standard laboratory mouse chow and tap water. Lights in the facility were set to go on and off at 12 h intervals with the lights going on at 7:00 a.m. In some experiments, mice also had *ad libitum* access to a high-fat diet containing 60% caloric content from fat (TD 06414; Harlan Teklad, Indianapolis, IN, USA) in addition to regular chow to measure dietary preferences. All procedures were approved by the Carleton University Animal Care Committee and followed the guidelines of the Canadian Council on Animal Care.

#### Effects of CF801 on Plasma Ghrelin Concentrations and Rebound Food Intake in Fasted Mice

Mice (*n* = 16) were singly housed and given free access to water and regular chow for a 4-day baseline period. After this, mice were assigned to one of three groups: vehicle (*n* = 5), CF801 low dose (*n* = 5; 11 μmol⋅kg^−1^ – LD), or CF801 high dose (*n* = 6; 22 μmol⋅kg^−1^ – HD). These mice were injected intraperitoneally once a day for 4 days starting at 10:00 a.m. Following 4 days of treatment, animals were tested for rebound feeding after a fast. To do this, we removed the food from each cage 1 h before lights out (6:00 p.m.), and food was returned to the cage the following day 1 h after the last injection. Food intake was then monitored every hour for 4 h to determine the effects of CF801 on rebound feeding. Animals were then sacrificed by rapid decapitation to collect trunk blood and other tissues. Two samples were lost during sample collection, so the number of samples used to measure acylated ghrelin was reduced to *n* = 14.

### Tissue and blood analysis

Following the treatment or recovery periods, mice were killed by rapid decapitation and plasma and tissue samples were collected. To measure glucose levels, glucose strips attached to a Contour glucose meter (Bayer Corp., Pittsburgh, PA, USA) were dipped in trunk blood collected before being centrifuged. The remaining trunk blood was collected in EDTA-coated tubes placed on ice and centrifuged at 800 × *g* for 15 min to separate plasma from red blood cells. Blood plasma was aliquoted separately to avoid multiple freeze/thaw cycles and stored at −80°C until processed. To protect the acylated ghrelin molecule, a 50 μL aliquot of blood plasma was treated with 2.7 μL of 1.0 N hydrochloric acid and 10 μL of 100 mM 4-(hydroxymercuric)benzoic acid prior to storage. Plasma acylated ghrelin for all animals was measured using an ELISA kit (Millipore). All samples analyzed had a coefficient of variation <10%.

#### Effects of CF801 on Food Intake, Weight Gain, and Adiposity

A second cohort of mice (*n* = 30) was acclimated to the laboratory conditions for 1 week prior to the onset of the experiment. Mice were housed individually and their body weight and food intake was recorded daily for a baseline period of 10 days. Given that ghrelin augments the intake of preferred diets (28), all mice received *ad libitum* access to a high-fat diet (60% of calories coming from fat), in addition to regular chow, to determine if CF801 altered dietary preference. Both chow and high-fat diet pellets were provided daily to mice *ad libitum* in pre-weighed amounts. Mice were then assigned to four different groups: non-injected control (NIC; *n* = 7), vehicle (0.9% saline) (VEH; *n* = 7), low-dose CF801 (11 μmol⋅kg^−1^ – LD; *n* = 8) and high-dose CF801 (22 μmol⋅kg^−1^ – HD; *n* = 8). Each day at 09:00 hours, mice were weighed and their food intake (both chow and high-fat diet) was recorded. After this, mice received an intraperitoneal injection of either CF801 or saline (except for the non-injected controls). Following the 13-day treatment period, half the animals in each group were sacrificed, while the remaining mice were allowed to recover from treatment. During the 8-day recovery period, mice were granted *ad libitum* access to standard laboratory chow, high-fat diet, and water, but did not receive any injections. At this point, the remaining mice were sacrificed. Carcasses from all animals were frozen and stored at −80°C until they were scanned for body composition analyses. Immediately prior to analysis, all carcasses were thawed to room temperature and weighed in order to obtain measures relative to total carcass mass. EchoMRI carcass analyses were performed at Health Canada, Nutrition Research Division facilities using the EchoMRI-1100 (System ID EF-020) scanner. Each carcass was analyzed separately and measurements of total fat mass, total lean mass, and total water weight were obtained for each mouse.

#### Effects of CF801 on Energy Expenditure

Mice (*n* = 10) were housed individually and their body weight and food intake was recorded daily for a baseline period of 10 days. As in Experiment 1, all mice had *ad libitum* access to regular lab chow and a high-fat diet (60% of calories coming from fat). At the end of the baseline, mice were housed in metabolic chambers (TSE Systems) for 48 h to examine metabolic rate using indirect calorimetry prior to the onset of the drug treatment. While indirect calorimetry measures were obtained throughout the 48-h period, we only analyzed measurements in the last 24 h to allow the animals to adjust to the chambers. Mice were then assigned to one of two groups: vehicle (0.9% saline) (VEH; *n* = 5) and CF801 (22 μmol⋅kg^−1^; *n* = 5). Each day at 09:00 hours, mice were weighed and their food intake (both chow and high-fat diet) was recorded, and then received an intraperitoneal injection of either CF801 or saline. Drug treatment continued for 16 days. On the eighth day, mice were again placed in the metabolic chambers for 48 h while still being monitored and injected with their respective treatment to determine differences in metabolism produced by the drug.

### Statistical analyses

In most experiments, group differences were analyzed using independent samples *t*-tests or one-way ANOVAs followed by Fisher LSD *post hoc* tests when significance was reported. Food intake and weight gain data over time were analyzed using repeated measures ANOVAs followed by *post hoc* one-way ANOVAs and Fisher LSD.

## Results

### CF801 inhibits acyl ghrelin secretion *In Vitro* and *In Vivo*

**Figure 1:**
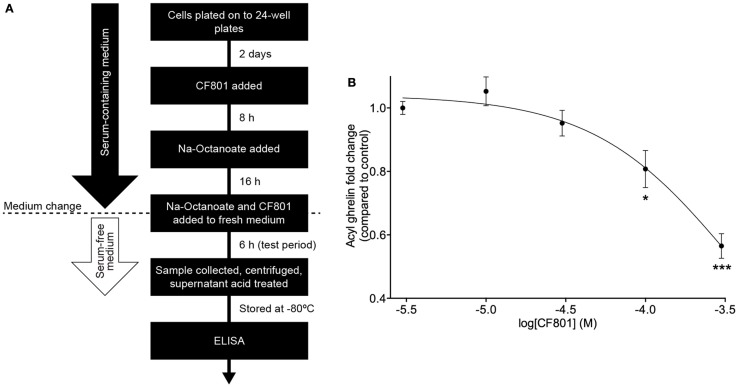
**CF801 inhibits acyl ghrelin secretion from SG-1 cells following 24 h of incubation**. **(A)** depicts the *in vitro* assay design. **(B)** shows Acyl ghrelin concentration in the culture medium collected during a 6 h test period following 24 h incubation at the indicated concentrations of CF801. All values are expressed as mean ± SEM. *p < 0.05, ***p < 0.001 significant difference in acyl ghrelin concentrations with CF801 treatment when compared to untreated control (*n* = 6 wells).

SG-1 cells are derived from stomach ghrelinomas induced by expression of the SV40 large T-antigen under the preproghrelin promoter. These cells retain many of the features of ghrelin cells within primary cultures of gastric mucosal cells from mice (61). They also show elevated expression of both preproghrelin and GOAT mRNA with high levels of acylated ghrelin synthesis and secretion (61). In order to test CF801’s ability to reduce acyl ghrelin secretion, we performed an *in vitro* cell-based assay using an SG-1 cell line as previously described (61, 62). No significant effects were found after a 6-h incubation period with CF801 (*p* > 0.05). However, following a 24-h incubation, CF801 inhibited acyl ghrelin secretion from SG-1 cells with an IC_50_ of 347 ± 40 μM, based on log-dose versus response non-linear fit. Significant decreases in secretion were found at concentrations of 100 μM (*p* < 0.05) and 300 μM (*p* < 0.05) CF801 (Figure 1).

**Figure 2:**
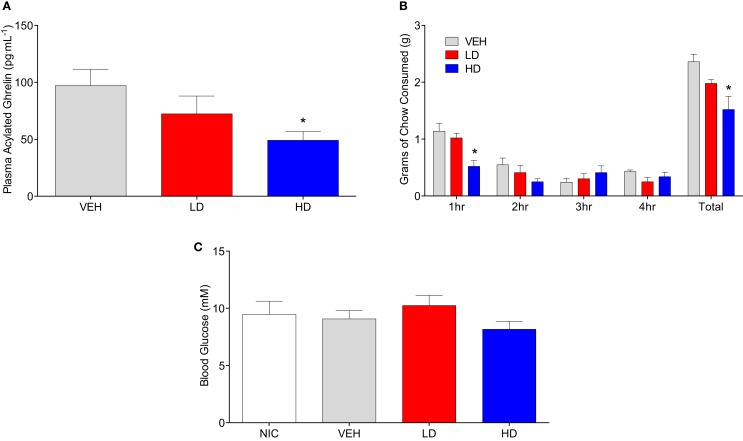
**Animals given HD CF801 displayed reduced levels of plasma acylated ghrelin following a 24 h fast compared to vehicle-treated animals (A)**. Average grams of regular laboratory chow consumed following a 24-h fast upon refeeding **(B)**, and blood glucose measured from trunk blood **(C)**. All values are expressed as mean ± SEM. *p < 0.05 relative to VEH-injected controls.

To determine if CF801 also altered acylated ghrelin concentrations, we conducted a study where mice were injected daily with saline or CF801 at one of two doses for 4 days. No effects of CF801 on food intake or weight gain were observed during the first 3 days of treatment (data not shown, *p* > 0.05). Before the last day of treatment, mice were fasted overnight, treated with their last injection, given access to food for 4 h and then sacrificed. Figure 2A shows plasma acylated ghrelin concentrations in mice treated with i.p. injections of saline, LD, or HD of CF801. As shown in the figure, CF801 caused a dose-dependent decrease in plasma ghrelin concentrations that was statistically significant [*F*_(2,24)_ = 2.90 *p* < 0.05, partial eta^2^ = 0.22] in mice treated with the high dose (*p* < 0.05 Fisher LSD). This decrease in acylated ghrelin was also associated with a lower amount of food consumed by mice treated with the high dose of CF801 during the first hour of refeeding [see Figure 2B; *F*_(2,24)_ = 2.394, *p* < 0.05, partial eta^2^ = 0.22, *p* < 0.05]. This was in correlation with acyl ghrelin concentrations (*r* = 0.492, *p* < 0.05). No significant effects were seen at any other time point examined (*p* > 0.05). Nevertheless, there was a significant overall dose-dependent treatment effect on the total amount of chow consumed during the 4-h period of food access after the fast [*F*_(2,24)_ = 4.527, partial eta^2^ = 0.411; *p* > 0.05]. This effect was statistically significant when examining differences in total chow consumed between the HD and saline treated groups (*p* < 0.01, Fisher LSD). No significant differences were observed between the groups in blood glucose concentrations [*F*_(1,15)_ = 1.105, *p* > 0.05; see Figure 2C].

### CF801 does not serve as an effective inhibitor of ghrelin octanoylation by GOAT

**Figure 3:**
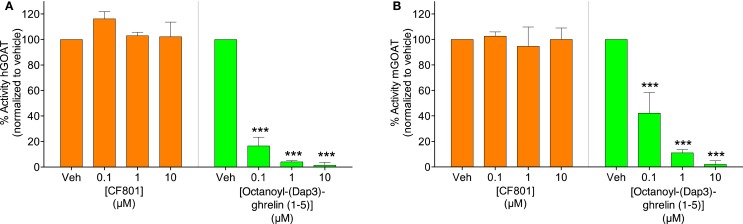
**Percent activity of human GOAT (A) and mouse GOAT (B) (obtained from membrane fractions from Sf9 cells expressing hGOAT and mGOAT, resepectively) when exposed to CF801 and octanoyl-(Dap3)-ghrelin (1–5)–NH_2_ using a fluorescence-based microsomal assay**. GSSFLC_AcDan_ was used as an acylation target with fluorescence measured by reverse phase HPLC with fluorescence detection. CF801 had no effect on either hGOAT or mGOAT acylation activity, while octanoyl-(Dap3)-ghrelin (1–5)–NH_2_ dose-dependently reduced GOAT activity. All values are expressed as mean ± SEM. ***p < 0.001 compared to vehicle control.

To test the potential that CF801’s ability to reduce acyl ghrelin secretion in SG-1 cells is due to direct inhibition of GOAT-catalyzed ghrelin octanoylation, we incubated CF801 with both the human and mouse isoforms of the GOAT enzyme under assay conditions previously described (63), with the potent GOAT inhibitor octanoyl-(Dap3)-ghrelin (1–5)–NH_2_ (56) examined in parallel as a positive control for GOAT inhibition. In this assay, we tested concentrations comparable with those reported for GO-CoA-Tat, which was reported to have an IC_50_ for GOAT of <1 μM (60). At concentrations up to 10 μM, no inhibition was seen with CF801 incubation [see Figure 3; hGOAT: *F*_(3,8)_ = 1.251, *p* > 0.05; mGOAT: *F*_(3,8)_ = 0.139, *p* > 0.05], while robust inhibition was seen with the control [IC_50_ of 20 ± 2 nM and 80 ± 10 nM with hGOAT and mGOAT, respectively; hGOAT: *F*_(3,8)_ = 485.023, *p* < 0.05, all doses less than vehicle with *p*’s < 0.05 by Fisher’s LSD *post hoc*; mGOAT: *F*_(3,8)_ = 51.476, *p* < 0.05, all doses less than vehicle with *p*’s < 0.05 by Fisher’s LSD *post hoc*]. This suggests that the observed effects of CF801 in cell- and animal-based studies are not caused by direct inhibition of ghrelin octanoylation by GOAT.

### CF801 treatment reduces high-fat diet intake and body weight gain without affecting caloric intake

**Figure 4:**
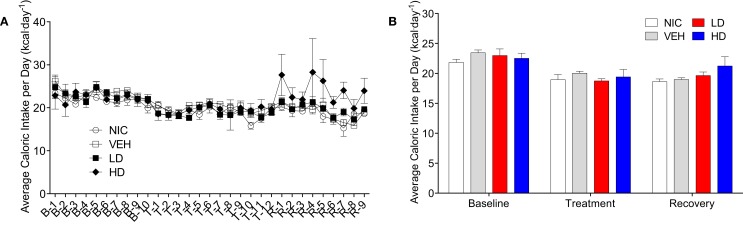
**Average daily (A) and overall average (B) caloric intake during the baseline, treatment, and recovery period**. All values are expressed as the mean ± SEM.

**Figure 5:**
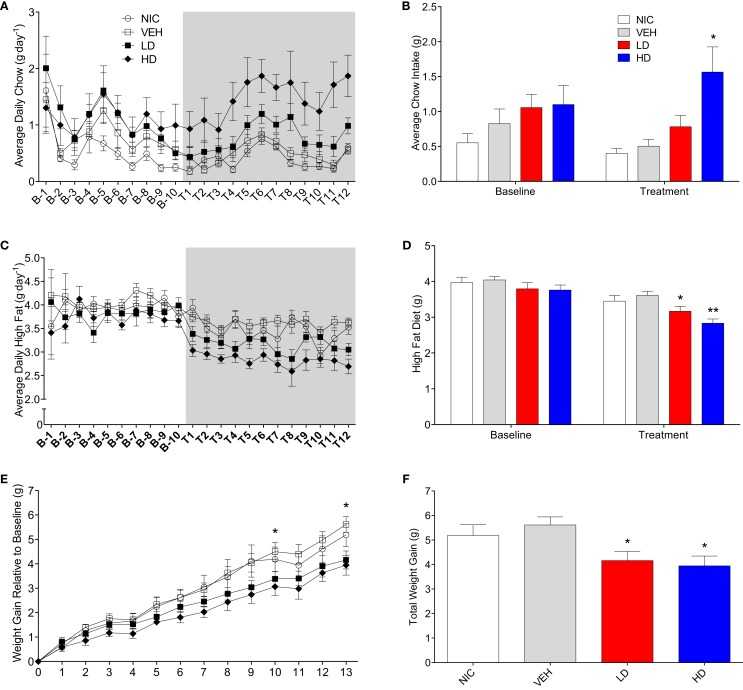
**Average daily (A) and overall average (B) chow consumption during baseline and treatment periods**. Average daily **(C)** and overall average **(D)** high-fat diet consumption during baseline and treatment periods. Weight gain since end of baseline **(E)** and total weight gain at the end of 13-day treatment period **(F)**. All values are expressed as means ± SEM. *p < 0.05, **p < 0.01 compared to VEH-treated controls, **(E)**: *p < 0.05 for HD and LD versus VEH.

Given that CF801 decreased ghrelin concentrations and rebound feeding, we decided to examine if CF801 affected weight gain and food intake in mice that had access to a high-fat diet in addition to regular chow. As illustrated in Figure 4, there were no differences between any groups in the amount of calories consumed during the baseline period [*F*_(1,29)_ = 0.586, *p* > 0.05], treatment period [*F*_(1,29)_ = 0.783, *p* > 0.05], and recovery period [*F*_(1,29)_ = 2.093, *p* > 0.05]. It was evident, however, that mice preferred the high-fat diet during the baseline period as they consumed a higher proportion of calories from this diet than the calories consumed from the regular chow diet (*p* < 0.05; see Figure 5). This diet preference changed during the treatment period. In particular, animals receiving high dose of CF801 consumed significantly more regular chow than animals in every other group as revealed by a one-way ANOVA followed by Fisher LSD *post hoc* [see Figures 5A,B; *F*_(3,28)_ = 5.305, *p* < 0.05, partial eta^2^ = 0.41]. In contrast, CF801 caused an overall significant dose-dependent decrease in the intake of the high-fat diet [Figures 5C,D; *F*_(3,29)_ = 6.788, *p* < 0.05, partial eta^2^ = 0.45]. There were no differences in high-fat diet consumption between NIC animals and VEH-treated animals. Animals receiving LD CF801 treatment consumed significantly less high-fat diet compared to both NIC and VEH groups (*p*’*s* < 0.05). Animals receiving HD CF801 consumed significantly less high-fat diet compared to both NIC and VEH groups (*p*’s < 0.05) and tended to consume less than animals receiving LD CF801 (*p* = 0.073). Interestingly, in correlation with decreased intake of the high calorie diet, mice treated with the high dose of CF801 showed a decrease in weight gain compared to mice treated with saline or mice that were not injected at all as determined by a one way ANOVA followed by Fisher LSD *post hoc* tests [Figures 5E,F; *F*_(3,29)_ = 3.098, *p* < 0.05, partial eta^2^ = 0.271]. Mice given the low dose of CF801 tended to have lower weight gain than control NIC (*p* = 0.092) and VEH-treated mice (*p* = 0.065), but this difference did not attain statistical significance. Nevertheless, *post hoc* tests also revealed that there were no differences in weight gain between mice treated with the low dose versus mice treated with the high dose of CF801 (*p* > 0.05).

### CF801 increases the utilization of fat as fuel

**Figure 6:**
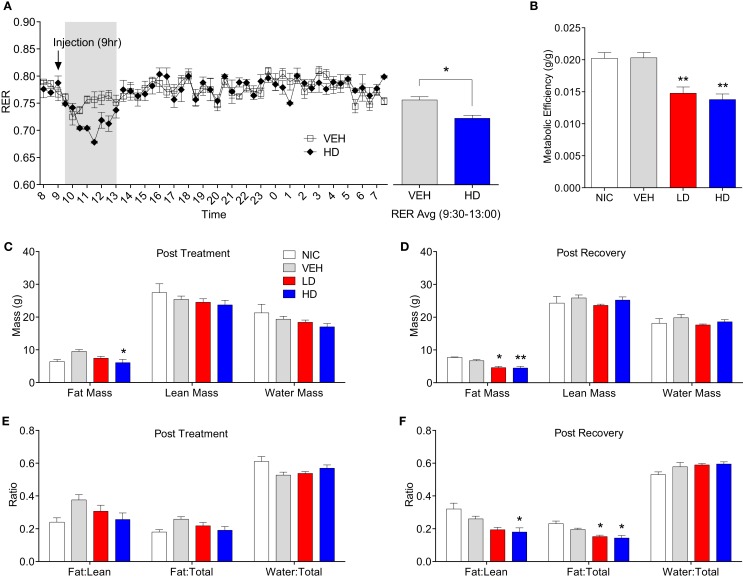
**Twenty-four hour respiratory exchange ratio following 8 days of vehicle and CF801 (high dose) treatment [(A), left] and average RER during the 4-h period following injection at 09:00 hours** [**(A)**, right]. Metabolic efficiency **(B)**; fat mass, lean mass, and water mass following treatment period **(C)** and recovery period **(D)**; fat mass:lean mass, fat mass:total mass, and water mass:total mass following treatment period **(E)** and recovery period **(F)**. All values are expressed as mean ± SEM. *p < 0.05, **p < 0.01 compared to VEH-treated animals.

Given that treatment with CF801 decreased weight gain without altering caloric intake, we reasoned that perhaps CF801 was exerting its effects at the metabolic level. To examine this, we examined differences in the respiratory exchange ratio (RER) of mice treated with the high dose of CF801 (*n* = 5) with that of saline-treated mice (*n* = 5) after a week of daily injections. While both groups of mice showed nearly identical RER during baseline period (*p* > 0.05), mice treated with CF801 displayed a significant decrease in RER during the 4-h period that followed the injection of the drug, an effect not seen in vehicle-treated mice [*t*(5) = 3.457, *p* < 0.05] (see Figure 6A).

### CF801 treatment alters body composition

Metabolic efficiency, defined as the amount of weight gained per calorie of energy consumed during a given time period, was calculated for each mouse during the baseline and treatment period. There were no differences in metabolic efficiency between any groups during the baseline period (data not shown). As illustrated in Figure 6B, there was a significant treatment effect on metabolic efficiency throughout the treatment period [*F*_(1,29)_ = 9.086, *p* < 0.05, partial eta^2^ = 0.522]. *Post hoc* analyses revealed that animals receiving LD CF801 had significantly lower metabolic efficiencies during the treatment period compared to both NIC (*p* < 0.05) and VEH treated animals (*p* < 0.05). Similarly, animals receiving HD CF801 showed significantly lower metabolic efficiencies during the treatment period compared with both NIC (*p* < 0.05) and VEH-treated animals (*p* < 0.05), but were no different than animals receiving LD CF801 (*p* > 0.05).

Given these changes in metabolic efficiency as well as the decrease in RER observed in the calorimetry experiment, we hypothesized that CF801 treatment would result in less accumulation of fat. To determine if this was the case, we conducted EchoMRI analyses on every animal post-mortem to identify differences in body composition as a function between groups. As shown in Figures 6C,D, the amount of total fat mass was significantly lower in mice treated with the high dose of CF801, compared to VEH injected animals right after the treatment period [*F*_(1,14)_ = 3.54, *p* > 0.05; partial eta^2^ = 0.515] and this decrease was more evident 2 weeks after the treatment period was terminated [*F*_(1,15)_ = 15.18, *p* < 0.05 partial eta^2^ = 0.805]. Mice receiving LD CF801 treatment and sacrificed at the end of the treatment period had lower fat mass compared to VEH-treated animals, but this was not statistically significant (*p*. > 0.05), but animals from the same group sacrificed 2 weeks after the last treatment had lower fat mass than mice in the VEH and NIC sacrificed at the same time point (*p* < 0.05). There were no differences between the groups in lean body mass or water mass (*p* > 0.05). Interestingly, there was a significant effect of treatment on fat mass:lean mass ratio following the recovery period [see Figures 6E,F; *F*_(1,15)_ = 7.735, *p* < 0.05; partial eta^2^ = 0.678]. An LSD *post hoc* analysis revealed that animals receiving LD CF801 treatment tended to show a reduction in fat mass:lean mass ratio following the recovery period compared to VEH-treated animals (*p* = 0.058) and had a significantly lower fat mass:lean mass ratio compared to NIC animals (*p* < 0.05). Similarly, animals receiving HD CF801 showed a significantly lower fat mass:lean mass ratio compared to both NIC animals (*p* < 0.05) and VEH-treated animals (*p* < 0.05), but were no different than animals treated with LD CF801 (*p* > 0.05). Furthermore, there was a significant treatment effect on the fat mass:total mass ratio following the recovery period [*F*_(1,15)_ = 9.245, *p* < 0.05; partial eta^2^ = 0.716]. LSD *post hoc* analysis revealed that both LD and HD CF801-treated animals had significantly lower fat mass:total mass ratios compared to NIC animals (*p*’s < 0.05) and VEH-treated animals (*p*’s < 0.05).

## Discussion

Given that ghrelin is one of the few peripherally circulating hormones capable of stimulating food intake, ghrelin has become a target for the pharmacological control of appetite and weight gain. Many attempts have been made at interrupting the ghrelin signaling system through the inhibition of ghrelin secretion (64), neutralizing the mature ghrelin protein via vaccination (65) or spiegelmer antagonism (66–68), and/or interrupting ghrelin signaling at the level of GHSR-1a (19, 21, 29, 41, 69). An emerging approach is the development of compounds that target GOAT as a potential therapeutic agent for combating weight gain and adiposity (56, 57, 59, 60). There are several advantages of targeting GOAT for the treatment of obesity, such as the selectivity to the targeted acylated ghrelin molecule, the uninterrupted constitutive activity of GHSR-1a (presumably necessary for appropriate growth hormone secretion) and the up regulation of unacylated ghrelin (UAG). Finally, it has been suggested that UAG may itself be a viable treatment option through counteracting the activity of acylated ghrelin and improving insulin sensitivity (70, 71).

In this paper, we show that CF801, a modified decapeptide similar in structure to the ghrelin peptide, reduces weight gain and adiposity without affecting total caloric intake. This is reminiscent of studies reporting that mice with targeted mutations to the gene encoding the message for the GOAT enzyme do not gain as much weight and store less adipose tissue when fed a high-fat diet compared to WT mice (46). Similarly, GHSR-*null* mice that do not respond to the acylated ghrelin product of GOAT enzymatic activity have reduced respiratory quotient and reduced metabolic efficiency, consume less food, and put on less weight, particularly in the form of adipose tissue, when given a high-fat diet (72). These findings are also similar to those from studies where mice were treated with GO-CoA-Tat, a previously characterized inhibitor of GOAT activity (60). Unlike GO-CoA-Tat, the mechanism underlying these effects does not appear to involve direct inhibition of GOAT octanoylation activity although it does involve a reduction in ghrelin secretion. Furthermore, CF801 was able to blunt the usual rebound eating response to a 24-h fast, reminiscent to what had been shown previously for a known GHSR antagonist (29).

One way in which CF801 could be decreasing weight gain and adiposity is through a dietary change. Indeed, while CF801 did not alter total caloric intake, it did affect the proportion of calories mice consumed from a high-fat diet and those consumed from laboratory chow diet. Looking at the baseline period, one can observe that mice generally preferred to consume the high-fat diet, a trend that continued throughout the study in control animals. CF801 treated mice, however, reduced their intake of the high-fat diet, and increased their intake of chow diet. It is unclear if this change in preference is due to an effect of the drug on preference or palatability, but it is certainly not due to a change in overall appetite as these mice ate enough to maintain their caloric intake throughout the study. It is important to note that at the end of the treatment period, there was a small spike in caloric intake in CF801-treated animals, suggesting that appetite increases once CF801 injections stop. This may indicate that CF801 is associated with enhancing signals that promote satiety, or simply by reducing ghrelin tone, but this needs to be studied further. The latter explanation is supported by data from our first study, where we show that mice repeatedly treated with CF801 decrease rebound food intake following an overnight fast in a dose-dependent manner and in correlation with acyl ghrelin concentrations in plasma.

Another way by which CF801 decreases weight gain and adiposity without affecting total caloric intake may be through a change in metabolism. It is well known that ghrelin favors the utilization of carbohydrates as a source of fuels as demonstrated by ghrelin-induced increases in RER in mice receiving daily injections of ghrelin (3). Here, we show that repeated injections of the higher dose of CF801 led to acute decreases in RER suggesting that CF801 changes metabolism favoring the utilization of lipids as fuel. This may also explain why CF801-treated mice also showed a lower proportion of body fat with no changes in lean or water mass. This is also similar to the effects of daily injections of GO-CoA-Tat, a treatment that also resulted in a reduction of overall fat mass relative to control vehicle-treated mice (60).

As described above, CF801 produces its effects through a reduction in acyl ghrelin concentrations, but not through a direct inhibition of GOAT activity. Using an *in vitro* cell-based assay, we were able to detect significant reductions in acyl ghrelin secretion from SG-1 cells following 24 h of incubation in 100 μM and 300 μM of CF801. Our preliminary data indicated that at a shorter incubation time of only 6 h, CF801 had no effect on acyl ghrelin secretion (data not shown), even at the highest concentration of 300 μM. Although Tat-peptide conjugates have been shown to attain maximal penetration within the first 3 h (73), our specific combination of amino acids has not been directly examined in this regard although time frames considerably above 3 h seem unlikely. Furthermore, stability of acylated ghrelin within vesicles is not known, but does not preclude the possibility of reserve acylated ghrelin packaged within secretory vesicles. Our data suggest that CF801’s effects may not be by direct interaction with the GOAT enzyme. This is also suggested by the lack of potency demonstrated by CF801 in the *in vitro* microsomal GOAT activity assay that directly assesses GOAT-catalyzed octanoylation of ghrelin mimetic peptide substrates. In contrast to the SG-1 cell-based assay and the effects of CF801 *in vivo*, CF801 was not able to reduce GOAT activity, while the known GOAT inhibitor octanoyl-(Dap3)-ghrelin (1–5)–NH_2_ showed a significant reduction.

Given the high amino acid similarity between CF801 and desacyl ghrelin, it is possible that CF801 acts like an analog of desacyl ghrelin. Indeed, desacyl ghrelin and the cyclic desacyl ghrelin analog AZP531 appear to have effects that are similar to those of CF801, including the decrease in weight gain, ghrelin secretion, and adipose tissue accumulation (74, 75). Alternatively, CF801 may reduce acylated ghrelin simply by reducing GOAT mRNA, and thus acyl ghrelin levels, without altering GOAT activity at least in the way that this is tested *in vitro*. While the mechanisms underlying these effects are not well understood, the idea that CF801 works like desacyl ghrelin or its analogs is an interesting contention as these are currently being proposed for potential clinical treatments to curb obesity. Given that ghrelin may be a hormone that is an important hormone in attenuating the effects of stress (19, 76, 77) or those of acute insults (78), more studies are required to determine if reducing acyl ghrelin concentrations with CF801 could have unwarranted side effects. Nevertheless, decreasing acyl ghrelin concentration peripherally with CF801 or with other bona fide GOAT inhibitors, may prove to be a desirable option for the treatment of obesity without affecting the functionality of the ghrelin receptor in the CNS.

## Conflict of Interest Statement

Alfonso Abizaid, Martin K. Wellman, and Zachary R. Patterson hold a patent on CF801 (WO/2015/010210). All other authors have nothing to declare.
